# Using metabolic energy to quantify the subjective value of physical effort

**DOI:** 10.1098/rsif.2021.0387

**Published:** 2021-07-21

**Authors:** Erik M. Summerside, Alaa A. Ahmed

**Affiliations:** ^1^ Neuromechanics Laboratory, Department of Integrative Physiology, University of Colorado Boulder, 354 UCB, Boulder, CO 80309-0354, USA; ^2^ Department of Mechanical Engineering, University of Colorado Boulder, 354 UCB, Boulder, CO 80309-0354, USA

**Keywords:** decision-making, effort, metabolic cost, movement control, cumulative prospect theory

## Abstract

Economists have known for centuries that to understand an individual's decisions, we must consider not only the objective value of the goal at stake, but its subjective value as well. However, achieving that goal ultimately requires expenditure of effort. Surprisingly, despite the ubiquitous role of effort in decision-making and movement, we currently do not understand how effort is subjectively valued in daily movements. Part of the difficulty arises from the lack of an objective measure of effort. Here, we use a physiological approach to address this knowledge gap. We quantified objective effort costs by measuring metabolic cost via expired gas analysis as participants performed a reaching task against increasing resistance. We then used neuroeconomic methods to quantify each individual's subjective valuation of effort. Rather than the diminishing sensitivity observed in reward valuation, effort was valued objectively, on average. This is significantly less than the near-quadratic sensitivity to effort observed previously in force-based motor tasks. Moreover, there was significant inter-individual variability with many participants undervaluing or overvaluing effort. These findings demonstrate that in contrast with monetary decisions in which subjective value exhibits diminishing marginal returns, effort costs are valued more objectively in low-effort reaching movements common in daily life.

## Significance

Nearly every action requires the expenditure of effort, yet the manner in which effort influences our decisions remains unclear. In movement decisions, effort is an inherent cost that when improperly valued may manifest in movement deficits such as the movement slowing seen in Parkinson's disease. Using a reaching task, we measured an objective representation of effort using metabolic cost, then had participants choose between reaching against different resistances to quantify how individuals subjectively value effort. We found that on average, effort is valued on a level that reflects the objective, metabolic cost. Furthermore, individuals are idiosyncratic in their valuation with an equal number undervaluing and overvaluing effort. These findings support a representation of effort as metabolic cost in models of decision-making and motor control.

## 1. Introduction

Economists have known for centuries that to understand an individual's decisions, we must consider not only the objective value of the rewards at stake, but their subjective value as well [1,2]. A nonlinear relationship is frequently observed between objective rewards and their subjective value, whereby individuals often value each additional increment of objective reward with diminishing subjective value (i.e. diminishing sensitivity). Understanding such nonlinearities has proven critical to our ability to explain decision-making across a range of economic environments and domains. However, every reward ultimately requires an action to obtain it, and that action inevitably requires effort. Effort is an inherent cost to many, if not all decisions, but we have yet to understand its role in decision-making. This is surprising, given that many neural disorders involve a form of movement deficiency.

One such example is Parkinson's disease. In Parkinson's disease, the cardinal symptom is bradykinesia, or slowness of movements. The disease arises from a loss of dopaminergic neurons in the substantia nigra. While dopamine is primarily thought to modulate reward signals, there is some evidence for its role in determining how hard humans and other animals will work for a given reward [3–6]. This implicates an altered cost/benefit valuation as one of the possible underlying mechanisms of movement slowing in Parkinson's disease [7].

One of the main obstacles to this line of research is the lack of an objective measure of effort costs. Psychophysical measurements demonstrate that the perception of effort increases nearly quadratically with increases in effort [8], suggesting that effort is valued in a similar manner. However, these studies, as well as previous attempts to understand how effort discounts reward in decision-making, have used indirect measures of effort such as isometric force production [9–15], estimated force production [16,17], number of targets acquired [18] and button presses [6,19]. So, we do not know if the objective effort costs were accurately represented by these experimental manipulations of effort. Some also required near maximum levels of exertion which may have led to additional costs such as pain, discomfort or fatigue. Others have provided choices coupled with monetary rewards without accounting for the accompanying nonlinearity in that reward's subjective value function [6,9,11–13,18]. We present a paradigm to circumvent these issues by examining low-effort movements that are representative of the everyday movements we make, controlling for reward, and critically, measuring objective effort directly in the form of metabolic cost.

Our understanding of movement control has a long history of implicating effort as an underlying determinant of preferred movement characteristics. In locomotion, effort costs are represented objectively as metabolic costs. Metabolic costs are the physiological energetic requirements involved in the body's conversion of chemical energy via substrate metabolism into chemical or mechanical work. In locomotion, metabolic costs have helped explain preferred walking speed, step length, step width and arm swing in healthy individuals [20–23]. When represented as metabolic cost, effort-based decision-making in reaching can account for both the choice of action and the vigour of the ensuing movements [24–27]. Metabolic costs are also used to explain foraging decisions in a range of animals [28–31]. Both the breadth and history of this literature provide a strong rationale to propose metabolic cost as an objective measure of effort.

Effort costs are also a cornerstone of optimal control models of movement control, which are capable of explaining observed movement trajectories across a range of conditions [32–34]. Such models invariably assume that the objective cost and the subjective valuation of the cost are one and the same. However, there is strong evidence that the subjective valuation of movement-related costs such as time and probability differ from their objective values [2,35]. When considering the subjective value of these costs, models of movement control can better predict movement-related behaviours [36–41].

Here, we will address two main questions regarding how physical effort costs are considered for effort-based decisions in healthy adults. First, is there a nonlinear relationship between the objective physical effort cost, quantified as metabolic cost, and its subjective value? Second, how does effort discount decisions? Together, these findings will help advance our understanding of the role of effort in both decision-making and movement.

## 2. Results

### 2.1. Measuring objective effort costs

**Figure 1.**
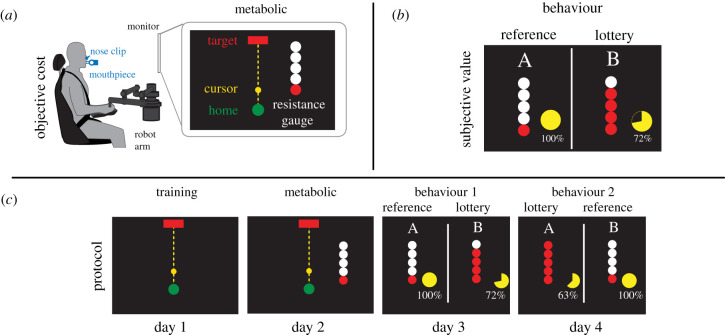
Experimental design. (*a*) Objective cost. Participants made reaching movements to move a cursor out to a target and back against a robot-generated resistance. In the metabolic sessions, participants wore a nose-clip and breathed through a mouthpiece in order to measure rates of oxygen consumption and carbon dioxide production. The scale of the force field was displayed on the screen as a resistance gauge that ranged from 1 to 5 dots with increasing red dots corresponding to increasing resistance. (*b*) Subjective value. Subjective valuation was calculated based on decision-making behaviour in behaviour session. Participants made decisions between reference and lottery options. The reference was a sure bet (100%) of reaching against the lowest resistance (1 dot) and remained constant throughout all trials. The lottery option was a combination of different resistance and probability pairs. The consequence of choosing the lottery was the risk of performing 5 min of reaching at the displayed resistance versus the alternative outcome of sitting quietly. (*c*) Protocol. In the training session, participants made constant duration reaches from a home circle (green) to a target circle (red) against an undisclosed resistance. In the metabolic session, participants reached against known resistances and learned to associate the displayed resistance gauge with the experienced resistance. In behaviour sessions 1 and 2, participants were instructed to choose which option (reference or lottery) they preferred.

**Figure 2.**
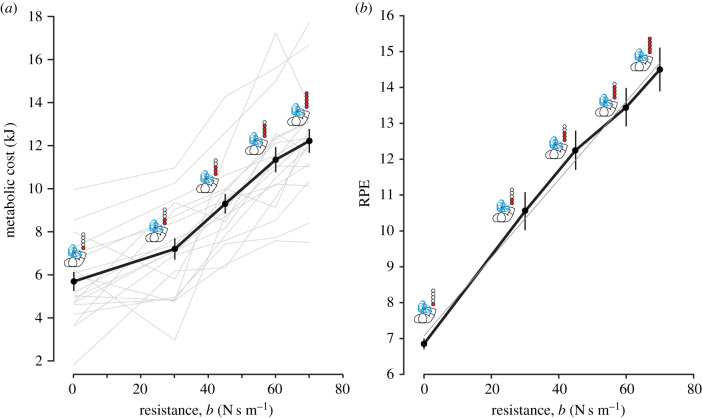
Objective effort cost (metabolic cost). (*a*) Metabolic cost (objective effort cost) increases with increasing resistance. Metabolic costs were measured at resistances of 0, 30, 45, 60 and 70 N s m^−1^. Data points shown are the average across participants for each resistance. Grey lines are individual participant data. (*b*) RPE increases with increasing resistance. Scores based on relative exertion to other experienced resistances. Thin line depicts linear fit to average data (*R*^2^ = 0.99, *p* = 0.0002). Error bars are s.e.m.

We quantified an individual's subjective valuation of effort as they performed effortful reaching movements. Participants performed out-then-back reaching movements against a resistive force and made decisions between a sure bet of having to perform low-effort reaches (reference option) or risk performing higher effort reaches (lottery option) (figure 1). Resistance was modulated according to the resistance (*b*) of a velocity-dependent force field. Effort was measured at resistances of 0, 30, 45, 60 and 70 N s m^−1^. The effects of additional costs such as time and accuracy were minimized, by strictly controlling movement duration and target size across conditions (see electronic supplementary material). The objective effort cost of each decision was quantified as the normalized net metabolic cost in joules (J) of reaching against each resistance for 5 min. Notably, metabolic cost was measured via expired gas analysis and thus represents a direct measure of effort cost. As resistance increased, metabolic cost exhibited a significant increase (figure 2*a*; *β* = 98.54, *R* = 0.70, *p* < 0.001). Movement duration and accuracy did not vary with condition (see electronic supplementary material).

### 2.2. Effort-based decision-making

To confirm that individuals perceived the differences in resistances across conditions, they reported their rating of perceived exertion (RPE) [42] for each resistance. Importantly, the range of this scale was limited to reflect solely reaching behaviour and as such, the numbers do not reflect conventional RPE scores. There was a significant increase in RPE that correlated with an increase in resistance (*β* = 0.109, *R*^2^ = 0.99, *p* < 0.001).

**Figure 3.**
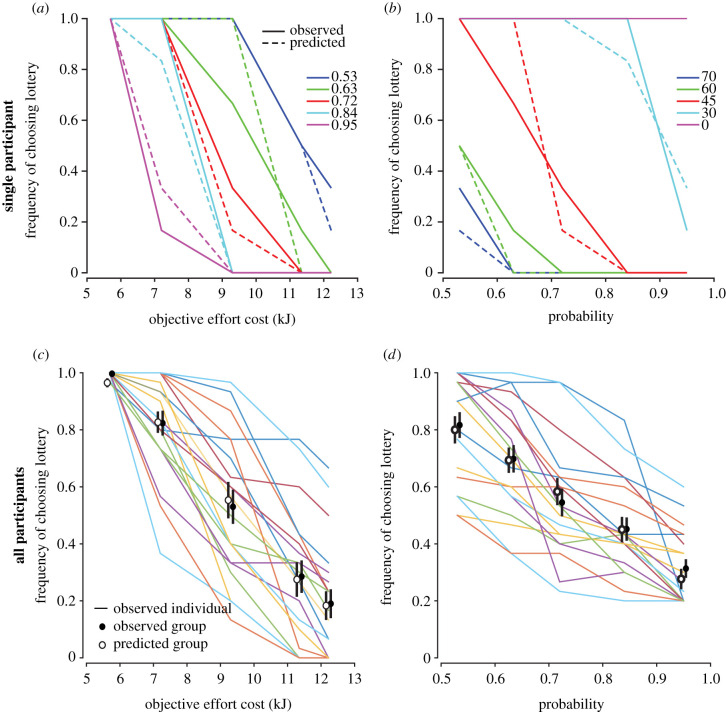
Choice behaviour and model predictions. (*a,b*) Single participant choice behaviour. Frequency of choosing the lottery based on objective effort cost (*a*) and probability (*b*)*.* Solid lines represent observed decision behaviour. Dashed lines represent model predictions based on fitted CPT parameters for SV model. (*c,d*) Frequency of choosing the lottery with increasing effort (*c*) and probability (*d*). (*c*) When collapsed across all probabilities, increasing effort led to a decreased frequency of the group choosing the lottery (filled circles, observed group mean). Predicted frequencies based on the fitted parameters of the SV model were able to successfully capture these preferences (empty circles, mean model predictions). (*d*) When collapsed across all effort levels, increasing probability of performing the lottery resulted in a decreased frequency of the group choosing that lottery. While the frequency of choosing the lottery tended to decrease with increases in either cost, the shape of these individual curves varied across individuals (coloured lines, observed behaviours of each individual).

To quantify each participant's subjective valuation of effort, they were asked to make choices between a reference option and a lottery option (figure 1*b*). The reference option consisted of a 100% probability of performing a low-effort reaching movement for 5 min. The lottery option consisted of either a known probability of performing a high-effort reach or the alternative outcome of sitting quietly for 5 min. Thus, in the reference option, subjects were assured of making a low-effort reaching movement, whereas in the lottery, there was the chance of making a high-effort reach or not to reach at all and sit quietly, depending on the outcome. In the lottery option, we varied the value of the reach probability and effort levels using combinations of one of the five resistances in combination with one of five probabilities (53%, 63%, 72%, 84%, 95%), for a total of 25 lottery combinations, repeated 6 times for a total of 150 trials. As the level of effort and/or probability increased in the lottery option, participants were more likely to choose the reference option, confirming that participants were considering both effort and probability when making their decisions (*Effort*: figure 3*a*,*c*, *β* = 0.00013, *Probability*: figure 3*b*,*d*, *β* = 1.19, *p*'s < 0.001).

Based on their decisions, we modelled their subjective valuation of effort using cumulative prospect theory (CPT) [2]. In this subjective value model, *M*_SV_, the expected utility, *E[U]*, of each option is determined as the subjective value of each outcome, SV(*x*), multiplied by its probability weighting, *ω*(*p*(*x*)):

2.1 E[U]=SV(x)ω( p(x)).

The subjective value SV(*x*) of each option consists of the effort cost:

2.2 $$\mathrm{SV}(x)=-x^{\alpha }.$$

The exponent *α* represents the nonlinearity between the objective cost, *x*, and subjective value of the effort expended, where *α* > 1 represents overvaluation of effort. Each additional increment of effort is valued with increasing sensitivity. Accordingly, *α* > 1 represents undervaluation of effort, and an *α* = 1 indicates an objective valuation of effort. The objective cost, *x*, is represented by the participants' average metabolic cost at each resistance level. The probability weighting function in CPT is modelled with a single-parameter s-shaped function

2.3 $$\omega (\;p(x))=\exp[-(-\ln{\left(\;p(x)\right)}^{\gamma })],$$

where *p*(*x*) represents the probability of the outcome and *γ* is a free parameter that determines the shape of the function. When probabilities are explicitly presented, as in this experiment, *γ* tends to have a value less than one, signifying that low probabilities are overweighted and high probabilities are underweighted [2].

The free parameter *α* was fitted using maximum-likelihood estimation. Participants exhibited idiosyncratic distortions in effort with an equal number either overvaluing or undervaluing effort. Notably, there was no correlation between an individual's effort sensitivity and their specific net metabolic cost of performing the task (*r* = − 0.0017, *p* = 0.9946).

**Figure 4.**
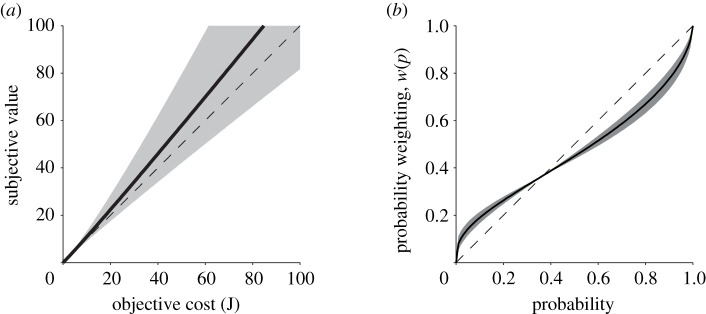
CPT parameter fits for SV model. Subjective valuation (*a*) and probability weighting (*b*) based on fitted *α* and *γ* parameters. Solid black line (grey shade) represents the mean (s.e.m.) across participants. Dashed black lines represent the lines of unity (*α* = 1, *γ* = 1).

While there was variability across participants, the fitted *α*'s on average were not significantly different from 1 (mean [95% CI], *α* = 1.037 [0.8675 1.2074], *p* = 0.65; figure 4*a*). This suggests that as a group, there was no consistent bias in the subjective valuation of effort costs required to complete the reaching task. Twelve of the participants returned for a second day of behaviour testing (figure 1*c*) and their decisions were largely consistent across days (see electronic supplementary material).

The second free parameter analysed was *γ*, which is a measure of how an individual weighted the probability of the risky decision. Eighteen of the 20 participants exhibited a *γ* < 1. The average *γ* across participants was 0.61 [0.418 0.801], which was significantly less than 1 (independent *t*-test, *p* < 1 × 10^−3^; figure 4*b*). Thus, most subjects overweighted small probabilities and underweighted large probabilities. This observation matches well with previous findings in similar tasks involving risky decisions [2,43,44]. As in effort valuation, participants' probability weighting was consistent across testing days (see electronic supplementary material).

Parameter fits to individual participants were validated by comparing model-predicted choices to each participant's choices. Similar to the behavioural data, as the effort cost of the lottery increased, the frequency of the model choosing the lottery decreased (*β* = 0.00012, *p* < 0.001; figure 3*a*,*c*). Also, as the probability of having to perform the lottery increased, the frequency of the model choosing the lottery decreased (*β* = 1.133, *p* < 0.001; figure 3*b*,*d*). Model-predicted choices were indistinguishable from actual choice data (linear mixed effects model, *Effort*: *p* = 0.742, *Probability*: *p* = 0.695).

Model performance in fitting participant choices was also compared to performance when fitting choices made by a random decision-maker. Comparison of the negative log-likelihood values confirmed that fits based on the participant choices were significantly better than fits to choices made by a random decision-maker (nLL_SV_ = 24.08 [19.07 29.09], nLL_random_ = 102.45 [101.89 103.02]). Individually, fits to each of the 20 participants outperformed fits to a random decision-maker.

It is possible that each individual's metabolic cost could better explain their decisions, compared to the average participant metabolic cost. To test this, we fit a model, *M*_ind_, where participant-specific choices were fitted using that participant's metabolic cost measurements. Otherwise, this model was identical to *M*_SV_, fitting the three parameters for effort valuation, probability weighting and temperature. This model, *M*_ind_, based on each participant's individual metabolic cost did not perform as well as the model using the participant average metabolic cost (nLL_ind_ = 29.11 [22.38 35.84]; nLL_SV_ = 24.08 [19.07 29.09]). However, we do find that the fitted parameters *α* and *γ* did not significantly differ between models (*α*: 1.15 [0.90 1.40], *p* = 0.31; *γ*: 0.55 [0.39 0.71], *p* = 0.38) and were also significantly correlated across models (*α*: *r* = 0.47, *p* = 0.04; *γ*: *r* = 0.48, *p* = 0.03).

### 2.3. Alternative effort valuation functions

To determine the significance of this distortion, we compared the full CPT model that considered each participant's fitted *α* and *γ* parameters to a control model that exhibited no distortion (*M*_lin_, *α* = 1):

2.4 $$SV(x)=-x^{1}.$$

We found that the model considering subjective valuation and probability weighting performed significantly better than a model considering solely distortions in probability (BIC_sv_ = −632, BIC_lin_ = −732; *p* < 0.001, Bayes factor; pxp_S_ = 0.9643, pxp_lin_ = 0.0351; table 1). On an individual basis, we found that 13 of the 20 subjects exhibited distortions in that a full model performed better than a reduced model without distortions (BIC_sv_ > BIC_lin_). Of these 13 participants, eight overvalued effort and the remaining five undervalued effort.

**Table 1.** Aggregate BIC scores and protected exceedance probabilities (pxp) for the models tested.

model	subjective value (*M*_SV_)	linear (*M*_lin_)	squared (*M*_2_)	hyperbolic (*M*_hb_)
no. parameters	3	2	2	3
aggregate BIC	−632	−732	−1311	−1517
pxp	0.9643	0.0351	0.0003	0.0009

We also investigated a different form of effort cost. We fitted a model where effort discounted utility additively, but effort costs were squared, *M*_2_ (*α* = 2):

2.5 $$\mathrm{SV}(x)=-x^{2}.$$

In contrast with *M*_sv_ and *M*_lin_, squaring the effort cost represents an overvaluation of effort that is consistent across individuals. We found that the model squared effort costs performed significantly worse than a model considering subjective valuation of effort costs (BIC_2_ = −1311; *p* < 0.001, Bayes factor; pxp_2_ = 0.0003; table 1). On an individual basis, we found that 18 of the 20 subjects exhibited distortions in that a full model performed better than a reduced model without distortions (BIC_sv_ > BIC_2_).

### 2.4. Alternative effort discount functions

It is also possible that our results are sensitive to the structure of the utility function. While the field of decision-making as a whole awaits conclusive evidence regarding the form of the discount function, there is much support for an additive utility in the literature [5,13,16,32,45–47]. Nonetheless, it has been proposed that effort, like time, discounts reward hyperbolically [14,48]. Therefore, we also investigated performance of a utility where reward is discounted hyperbolically by effort:

2.6 $$\mathrm{SV}(x)=-\frac{1}{1+x^{\alpha }}.$$

However, this hyperbolic model significantly underperformed a model in which utility is the sum of reward and effort costs (BIC_hb_ = − 1517; *p* < 0.001, Bayes factor; pxp_hb_ = 0.0009; table 1). The additive utility performed better in 17 out of 20 participants.

## 3. Discussion

Here, we quantified subjective valuation of effort in a moderately effortful movement task. Using a novel approach, we considered objective effort to be represented by the metabolic cost required to perform the movement, and explicitly measured that metabolic cost via expired gas analysis. Our protocol used a risky decision-making task that allowed us to map utility directly onto effort in the appropriate units of energy (joules) without the confound of intermediate conversions such as money, force, number of movement repetitions and time. As such, our approach provided us with new and powerful insights into how effort is truly represented in movement tasks, and mitigated the inherent inaccuracies and approximations in other approaches that are less naturalistic and use less ecological representations of effort. Therefore, these results provide a unique window into how physical effort is considered when choosing between effortful movements.

With increasing effort costs, are additional increments in objective effort overvalued or undervalued? We found on average, participants valued effort objectively. While there was a significant nonlinearity in the relationship between effort and its subjective value, the shape of this nonlinearity varied idiosyncratically across subjects, with no consistent distortion observed. Approximately an equal number of participants overvalued and undervalued effort, with the group average indicating an objective valuation of effort, highlighting extensive inter-individual variation in effort valuation across individuals. Despite the large variability across individuals, this function remained fairly robust within an individual across testing days.

Only recently have scientists begun to probe the effects of effort costs on decision-making. Previous work delving into physical effort has tended to focus on how effort costs discount reward, producing an overall utility for each prospect [5,13–16]. Candidate utility functions have been proposed that take either a hyperbolic or quadratic shape. The use of a hyperbolic function stems from the idea that effort discounts reward in the same manner that time discounts reward. However, there is no conclusive evidence supporting such a function. Moreover, we find that a model of utility in which reward is discounted hyperbolically by time performs significantly worse in explaining subject choices. The quadratic shape is equivalent to setting the parameter *α* = 2 in our analysis. This shape was derived on the premise that the subjective value of effort costs increases supralinearly, drawing from findings in the perception literature [49,50]. Our results demonstrate that in the case of moderately effortful tasks, effort sensitivity does not consistently increase supralinearly across participants.

There are a few possible explanations for the apparent discrepancy between our findings and those of recent studies [13,15]. First, we probed a lower range of effort requirements. Both Hartmann *et al*. and Klein-Flugge *et al*. probe effort levels up to approximately maximum effort. Our goal was to identify effort valuation in moderately effortful tasks similar to those experienced in common daily activities. It is possible that the nonlinearities previous studies observe in effort valuation may only begin to emerge only at near-maximal effort levels as a result of pain, discomfort, injury risk or force saturation effects.

Another difference between our study and previous ones on effort-based decision-making is the influence of subjective valuation of reward. Many studies make the assumption that the subjective valuation of the reward increases linearly with an increase in reward magnitude. Levy *et al*. [51] found that different rewards including money, food and water are all valued nonlinearly. To minimize the possible confound of subjective reward values, our paradigm was designed in a manner void of explicit rewards. Participants were instructed to make decisions based solely on effort expenditure. While monetary compensation was provided to all participants for completing the experiment, it was distributed equally and independently of choice behaviour.

Prior investigations have also used less direct measures of effort modulation rather than directly measure metabolic cost. Common approaches have included grip-force, number of buttons pressed and sizes of obstacles scaled [13,15,49,52]. While metabolic cost is likely to increase in all these cases, the shape of the relationships has not been identified. As such, any nonlinearities observed may be a result of a nonlinear mapping between metabolic cost and the proxy employed. A novel method introduced in our study is that we measured changes in effort based on the amount of metabolic energy used to perform each task, allowing us to directly quantify the relationship between the objective and subjective costs of effort.

Other studies have examined the role of physical effort in reaching movements, without the confound of monetary rewards. A recent study by Morel *et al*. [16] observed a near-quadratic sensitivity to effort, even for a range of low-effort values, when effort was quantified as the resistive force. Another study [17] also reported overvaluation of effort, when effort was quantified as resistive force. One possible reason for the discrepancy in our current findings is that in both these studies, effort was represented as resistive force, rather than metabolic cost. Here, we show that metabolic cost actually increases slightly supralinearly with resistive force. Indeed, we found that while metabolic cost increased with resistance, the relation between metabolic cost and resistance was slightly better fitted with a quadratic, than with a simple linear fit (quadratic: *mc* = *a* + *b*(resistance)^2^, *R*^2^ = 0.97, *p* = 0.0018; linear: *mc* = *a* + *b*(resistance), *R*^2^ = 0.95, *p* = 0.0038; figure 2*a*). Thus, a nonlinear mapping between metabolic cost and resistive force could help explain the greater effort sensitivity observed in earlier studies.

Similar to other effort paradigms, we added a probability cost. Probability was necessary to make lottery combinations that were similar in subjective value to the reference option. To account for known distortions in probability weighting, we used a single-parameter Prelec function [53]. Little is understood about how this parameter behaves in losses, but when comparing our results to a similar function originally proposed by Tversky & Kahneman [2], we find qualitatively similar and statistically indistinguishable results [2,43,44]. This consistency strengthens our conclusion on effort valuation by considering the effects caused by distortions in probability weighting.

The theoretical framework of optimal feedback control has significantly advanced our understanding of movement control [54]. A key component of such models is the incorporation of a cost function that includes an effort cost. The effort cost has historically been represented as the sum of the squared forces or squared motor commands required to generate the movement. The quadratic term is largely due to mathematical convenience, since experimental results in both humans and other animals performing isometric force tasks have shown that effort costs, measured as metabolic cost, align more closely with the integral of absolute force, not squared force, over time [55]. One possible justification for the quadratic term is that while effort costs increase linearly with force, the subjective value of effort may increase supralinearly with force. However, our results demonstrate that on average, effort costs are valued objectively, and suggest that these cost functions should consider this in order to more accurately represent objective effort costs.

Recent models of decision-making and movement control predict that as the effort requirements of a movement of a given distance increase, the speed with which that movement is executed should decrease [7,24,34,46]. Indeed, behavioural findings have confirmed this prediction in reaching tasks [56]. Work by Mazzoni *et al*. [7] suggests the slower reaching speeds observed in Parkinson's patients is a result of an exaggerated cost/benefit ratio. Following up on these observations, it would be interesting to determine whether differences in sensitivity to effort costs in a healthy population could explain inter-individual variability in preferred movement speeds.

### 3.1. Limitations

During the decision-making aspect of our experiment, a number of red dots ranging from 1 to 5 were presented on the side of the screen. Despite heavy practice reaching under each resistance, it is possible that some participants could have interpreted the linearly increasing dots as representing a linear increase in force. However, the resistive forces represented by these dots did not increase linearly. The nonlinearity in increasing force allowed us to probe whether individuals were associating effort based on the resistance itself or rather the number of dots. To differentiate between these two strategies, individuals report an RPE after making movements at each condition. While our results suggest that participants were able to properly sense the changes in resistance (linear scaling of RPE with resistance), there is still a possibility that the scaling of the cues may have influenced the representation of effort. Using a scaling based on the dots in this experiment would result in an overvaluation of lower effort and an undervaluation of higher efforts, a result counter to what is predicted in a quadratically increasing effort cost.

The costs of accuracy and time play an essential role in forming the utility of a movement. In the current protocol, we aimed at controlling both of these costs in several ways. To control for accuracy, we used a very large target (15 cm diameter) such that any deviation from the centre was inconsequential to movement success. Results found in the electronic supplementary material (Performance Measures) reaffirm that accuracy in terms of speed and crossing-point deviation did not covary with increasing effort. Furthermore, the constrained movement durations were identical across all effort conditions such that any cost of time would be independent of the level of effort tied to the lottery. It is possible that the cost of time associated with sitting quietly may be valued differently from the cost of time while engaged in activity [57]. An increased cost of sitting quietly may have led participants to choose the reference more frequently than if this potential cost of waiting was removed. To address this, we fitted a model that includes the cost of sitting and found that it did not explain behaviour better than a model without the sitting cost (see electronic supplementary material, ‘Alternative Models’). Nonetheless, failure to account for this potential cost would mean that our function is over-representing effort, a finding that would further argue against the commonly believed quadratic shape of the effort discounting function.

Our findings demonstrate that there are idiosyncratic distortions in an individual's sensitivity to effort costs in a low-effort task, with some individuals showing increasing sensitivity to effort and yet others exhibiting diminishing sensitivity. However, on average, individuals valued effort objectively, in contrast with previous observations of a quadratic valuation. Together, these findings provide the first quantification of effort valuation in reference to an objective physiological effort cost, and reveal an objective valuation in low-effort reaching tasks representative of activities of daily life.

## 4. Material and methods

Twenty participants were enrolled in this experiment (age: 25.35 ± 4.00 years, weight: 72.90 ± 9.21 kg, 7 females). Each participant gave written informed consent as approved by the University of Colorado Institutional Review Board and received $10 h^−1^ for participating. All participants completed a training session, metabolic session, and one choice behaviour session. Twelve repeated a second choice behaviour session. One participant's metabolic data were corrupted and removed from the metabolic analysis.

### 4.1. Training session

The purpose of this session was to familiarize participants with reaching against a resistive force as well as to train them to reach under a constrained time limit. The task consisted of making 20 cm out-then-back reaching movements between a home circle and a rectangular target (15 cm wide). Visual feedback was provided at the end of each movement to ensure that movement duration fell between 550 and 650 ms. Velocity-dependent forces were generated according to the following equation: [*F_x_ F_y_*] = − **b**[*V_x_ V_y_*], where *F_x_* and *F_y_* represent horizontal and vertical forces, *V_x_* and *V_y_* the corresponding handle velocities and **b** is a constant describing the scaling of the resistance. There were five conditions: **b** = 0 (no forces), 30, 45, 60 and 70 N s m^−1^, each presented twice in blocks of 50 trials in randomized order.

Immediately following each block in the training session, participants reported a modified RPE where they were asked to rate the physical effort required to complete the task. The first block tested was 0 N s m^−1^ followed by the second block tested at 70 N s m^−1^. By providing these two blocks early, participants were able to set a floor and ceiling score to base the remaining three conditions within. RPE scores did not represent absolute levels of exertion, but instead levels of exertion relative to an already experienced maximum (70 N s m^−1^) and minimum (0 N s m^−1^) resistance. After each block, participants completed 20 washout trials against no resistance (0 N s m^−1^). Each resistance condition (sin null resistance) was repeated three times for a total of 13 blocks. The last block of each condition was inspected to confirm that the increase in resistance reflected a relative increase in RPE score.

### 4.2. Metabolic session

In the metabolic session, the objective effort cost (i.e. metabolic cost) of reaching against resistance was measured using methods previously developed in our laboratory [58,59]. Upon arrival at the laboratory, participants completed three 6 min baseline blocks where they sat quietly and we measured their metabolic cost. This was followed by trial blocks of reaching against resistances of 0, 30, 45, 60 and 70 N s m^−1^. Robot force, position and velocity were recorded at 200 Hz. The resistance in each block was fixed, but the order of blocks was randomized. Each reaching block consisted of 300 trials lasting approximately 7 min. Expired gas analysis was used to calculate the net metabolic cost (J) of seated resting and of seated reaching against resistance. Before participants arrived for the metabolic session, they were instructed to refrain from eating and drinking anything but water for the morning of the session. Fasting was necessary to minimize the thermal effect of food on basal metabolic rate [60]. Participants wore a nose-clip and breathed into a mouthpiece during all baseline and reaching blocks (figure 1*a*). A metabolic cart (ParvoMedics, TrueMax2400) was used to sample the amount of consumed oxygen (O_2_, l min^−1^) and expired carbon dioxide (CO_2_, l min^−1^) over 5 s intervals. At the beginning of each metabolic session, gas fractions were calibrated to within an error of ±0.03% using a certified standard gas mixture and flow rate was calibrated to within an error of ±0.2% using a 3 l syringe under various flow rates. Respiratory exchange ratio (CO_2_/O_2_) was monitored to confirm that each subject was within physiological ranges of aerobic respiration (0.7–1.0). Using the average O_2_ and CO_2_ for the last 4 min of each reaching condition, gross metabolic power (J s^−1^) was calculated using the Brockway equation [61]. Only the last 4 min of reaching were analysed to account for both physiological and equipment delays. The primary source of physiological delays is a result of the necessary time for the expired metabolites to accurately represent the metabolic activity in the active muscle. The primary source of equipment delay is due to the time required to expire the ambient air through the system. The net metabolic power for each condition was calculated by subtracting the lowest average gross power of the three baseline blocks from the average gross metabolic power of that condition. The average net metabolic power measured for each resistance was multiplied by the duration of the activity to represent the net metabolic cost of reaching against that resistance (J). We refer to the net metabolic cost of reaching as the objective effort cost.

For each reaching block, there was a gauge on the side of the monitor displaying a number of red dots ranging from 1 to 5 (figure 1*a*). The number of dots corresponded to the magnitude of the resistance (1 dot = lowest resistance, 5 dots = highest resistance). Participants were instructed to associate the dots with the resistance they were experiencing. They were also informed that the increase in resistance did not necessarily scale linearly with the number of dots. These dots were used in the upcoming behaviour session to represent effort levels. Between each reaching block, the participant rested for 5 min.

### 4.3. Behaviour sessions 1 and 2

In the behaviour sessions, participants made choices between risky effort lotteries. Based on their choices, we determined their subjective valuation of effort.

#### 4.3.1. Choices

Participants were instructed to choose which of two reaching options they preferred. In each pair of options, one was a reference option and the other was a lottery, with each option involving the performance of an effortful reach continuously for 5 min. The reference option was presented in every trial as a zero resistance reach (0 N s m^−1^; figure 1*b*). Throughout the session, the reference option was consistently displayed on one side of the screen, either the left or right. The side was randomly determined for each subject. The lottery option consisted of a probability of performing a high resistance reach with the alternative outcome of sitting quietly. The lottery was presented with a percentage value (53%, 63%, 72%, 84%, 95%) and a given resistance communicated by the number of red dots (figure 1*c*). The percentage represented the chance of having to reach continuously for 5 min and the number of dots represented the resistance experienced. The alternative outcome was to sit quietly in the chair for 5 min. Importantly, each potential outcome had the same duration to control for the effect of time. A single block of choice trials consisted of each resistance being paired with each percentage for a total of 25 reaches per block. The behaviour session consisted of six continuous blocks with the order of each block being randomly generated for a total of 150 choice trials. Each choice combination was presented on the screen for 4 s then the combination disappeared and the participant had 2 s to record their choice. Decisions were recorded by pushing one of two buttons on a remote control. No information was provided regarding previous choices and no actual reaching was performed between choices.

#### 4.3.2. Realization

At the end of the behaviour session, the participants' choices were realized by performing the results of three randomly chosen choice trials. If the subject chose the reference option, then they performed 5 min of reaching against zero resistance. If the participant chose the lottery option, they rolled two 10-sided dice. If the number rolled was higher than the percentage of the chosen lottery, the participant would sit in the chair for 5 min and not have to perform the reaching task. Otherwise, they performed 5 min of reaching against the resistance assigned to the lottery.

### 4.4. Quantifying subjective valuation and probability weighting

The average metabolic cost (objective effort cost) measured across participants in the metabolic session was combined with individual choice behaviour in the behaviour session to calculate each participant's specific utility function for effort. Their choices were used to fit decision-making parameters found in utility functions derived from CPT [2] using maximum-likelihood estimation.

The first model examined, *M*_sv_ , considered a utility in which the subjective value of effort was subtracted from utility:

4.1 $$\mathrm{SV}(x)=-x^{\alpha }.$$

The parameter *α* is a measure of the subjective valuation of the effort cost. There is no explicit reward in the task, so we assume reward is zero and the utility is entirely determined by the subjective value of effort. The effort cost, *x*, was represented as the net metabolic cost with respect to quiet sitting. As sitting quietly was the baseline condition to which the effort of each reaching condition was compared, the net effort cost in the seated condition was 0. The second parameter, *γ*, determines the shape of the probability weighting function, *ω*(*p*(*x*)):

4.2 $$\omega (\;p(x))=\exp[-(-\ln{\left(\;p(x)\right)}^{\gamma })].$$

Together, these two functions determine the option's expected utility (*E*[*U*]):

4.3 E(U)=SV(x)ω( p(x)).

In a two-alternative forced choice task, the *α* and *γ* variables are fitted to the observed behavioural data using the following function, where the subscripts *L* and *R* refer to the lottery and the reference options, respectively:

4.4 $$P_{L}=\frac{1}{1+e^{k(E[U_{R}]-E[U_{L}])}}.$$

*P_L_* represents the probability of choosing the lottery and is based on a logistic function that considers the difference in EU of each choice as well as the third free parameter,  *k*, which describes the logistic slope. All three parameters were fitted using the maximum-likelihood estimation. Parameter fits were confirmed by using maximum-likelihood calculations from over 1000 model runs using different initial conditions to minimize the risk of settling on a local minimum. Additionally, we used negative log likelihood to compare model performance to that of a random decision-maker, as well as a model based on fitting individual metabolics to individual choices, rather than group average metabolics to individual choices.

We also tested a linear discount function, *M*_Iin_, in which effort is valued objectively:

4.5 SV(x)=−x.

Here, *α* was fixed to 1, and *γ* and the temperature, *k*, were free parameters. Based on previous findings in both the perception and decision-making literature, we tested an additive model, *M*_2_, where effort was squared:

4.6 $$\mathrm{SV}(x)=-x^{2}.$$

Here, *α* was fixed to 2, and *γ* and the temperature, *k*, were free parameters. This function implies that effort is consistently subjectively valued quadratically, meaning that small increases in effort are valued as less costly compared to larger increases in effort, leading to a concave discounting of reward.

We also considered a hyperbolic effort discount function in which utility was discounted hyperbolically by effort, *M*_hb_. Here, the subjective value of the option is represented as

4.7 $$\mathrm{SV}(x)=-\frac{1}{1+x^{\alpha }}.$$

Goodness of fit of the full additive model which considered subjective value, *M*_SV_ , was compared to the alternative models, M_*_, using Bayes factors (BF):

4.8 $$\mathrm{BF}=\frac{\;p(M_{\mathrm{sv}}|D)}{\;p(M_{*}|D)}=\frac{\;p(D|M_{\mathrm{sv}})P(M_{\mathrm{sv}})}{\;p(D|M_{*})P(M_{*})}.$$

Bayes factors were approximated with the Bayesian information criterion (BIC) [62], calculated as the log likelihood of the model with the best fit parameters, minus a penalty for the number of parameters, *n*, and datapoints, *m*:

4.9 $$\mathrm{BIC}=\log(\;p(D|M,{\hat{\theta }}_{M}))-\frac{n}{2}\log m\approx \log(\;p(D|M)).$$

BICs and Bayes factors were calculated for each subject, each model and each model comparison. Aggregate BICs are presented as well as population-based Bayes factors, which were interpreted as *p*-values with the following adjustment: *p* = 1/BF [63]. Finally, we also present the protected exceedance probabilities (pxp) [64,65] using the spm_BMS function available in SPM12 software (Wellcome Trust Centre for Neuroimaging, London, UK; http://www.fil.ion.ucl.ac.uk/spm).

### 4.5. Movement speed

*Metabolic session:* From the movement data acquired during the metabolic session, we calculated the average and peak tangential velocity and movement error for each trial. Movement error was calculated as the absolute lateral deviation of the cursor from the centre of the target when it had reached a distance of 20 cm from the centre of the home circle. We compared the average velocity in each block (i.e. resistance condition) to determine whether participants had consistently maintained the required movement velocity across resistance conditions. A similar analysis was performed for movement error.

### 4.6. Statistics

The effect of resistance on metabolic cost, RPE and frequency of choosing the lottery (both observed and model) was quantified using a simple linear regression model. The parameters *α* and *γ* were compared to unity using independent *t*-tests. Comparing the observed frequency of choosing the lottery to the model frequency of choosing the lottery was performed with a linear mixed effects model in both effort and probability comparisons. In these comparisons, a dummy variable was used to indicate whether behaviour was observed or modelled. The analysis of the effect of resistance on reaching velocity and movement error was performed with a linear mixed effects model. All statistical analyses were conducted using a significance level of 0.05. Unless otherwise noted, descriptive statistics are presented as mean [95% confidence interval].

## Data accessibility

Choice and metabolic cost data are available from the OSF repository: https://osf.io/3x9ur/. The data are provided in electronic supplementary material.
